# Dual Goals, Dual Agency: The Perils of Measurement and Control: Comment on "Dual Agency in Hospitals: What Strategies Do Managers and Physicians Apply to Reconcile Dilemmas Between Clinical and Economic Considerations?"

**DOI:** 10.34172/ijhpm.2022.6764

**Published:** 2022-04-12

**Authors:** Henrich R. Greve

**Affiliations:** INSEAD, Singapore, Singapore.

**Keywords:** Organizational Goals, Multiple Goals, Self-enhancement, Goal Compromise

## Abstract

This commentary to Waitzberg et al draws on the research stream on organizational goals in management to examine the findings they report, point out the correspondence of their findings and interpretation with existing theory, including development beyond it. Their work discusses these considerations very well. It also suggests paths to further theoretical development and proposes how their work demonstrate the potential for further research on multiple goals in hospitals. Such research will be important both for health policy and management and for management theory and practice generally.

## Background

 Waitzberg et al have provided important evidence on how hospital professionals seek to resolve dilemmas as they pursue their often-conflicting clinical and economic goals, and the theoretical development they draw from this evidence is compelling.^1^ In the following commentary, I make additional remarks that connect this research to central issues in the management of organizations with multiple goals,^2^ discuss the importance of self-enhancement theory, and call for further research on this topic.

## Measurement of Objectives

 A central feature of management practice is the assignment of goals to organizational units and the individuals who manage them. There is significant research showing that the common response is to search for solutions when falling short of a goal, and to remain unchanged when reaching a goal.^3,4^ Two features of this process are often taken for granted, and hence see insufficient research attention: First, organizational goals control behaviors much more strongly when they are turned into numeric measures.^5,6^ Second, numeric goals set to specific levels control behaviors more strongly than vague “do your best” instructions.^7^

 Both features of the goal-setting process are important for hospitals because there is significant variation in the degree to which numeric performance measures are collected and reported, and goals are assigned and compared with the actual performance. Clinical care results in income, incurs costs, and has variable recovery rates and durations. Who is exposed to this information and asked to manage according to it? Who is not exposed to it, but instead is exposed to managerial instructions to reduce use of expensive equipment and personnel and accelerate treatment schedules, all while producing good clinical outcomes? As Waitzberg et al report, there is significant variation in how hospitals distribute information about actual economic outcomes among the clinical personnel. How does this shape their relationship with management?

## Multiple Objectives

 Finding the answer to this question requires consideration of how multiple goals affect organizational decision-making. Organizational goals are sometimes in conflict and sometimes in agreement, but they will always be viewed as separate ways of assessing the performance of an organizational unit. As a result, multiple goals potentially prevent organizational change because an individual can engage in self-enhancement by emphasizing one goal that shows high performance in order to forestall changes to solve problems indicated by another goal that shows low performance.^2^ For organizations that have multiple goals of equal or similar importance, this is a dilemma that raises the question of whether assigning multiple goals to the same decision-maker might be less effective than having different decision-makers who each specializes in one goal. Self-enhancement theory predicts that clinical personnel assigned both clinical and economic goals could neglect both kinds of goals.^2,8^ Clinical personnel assigned clinical goals and managerial personnel assigned economic goals may perform better if they can use negotiation and teamwork to address whichever goal was problematic.

## Application of Self-enhancement Theory

 The findings reported by Waitzberg et al suggests that the sampled clinics did in fact have some problem-solving patterns typical of organizations in which decision-makers face goal measurement and multiple goals, but with a focus on different goals depending on the type of decision-maker. Consider the three categories of behaviors they documented: (1) increase efficiency, (2) reshape management, and (3) reframe decision-making. The first of these is straightforward, as efficiency increases that do not worsen clinical outcomes represent successful problem solving. Indeed, it is interesting, and consistent with the theory, that the evidence presented by Waitzberg et al suggests that interactions between personnel with economic goals and personnel with clinical goals triggered some of the reported improvements.

 Reshaping management shows similar behavioral outcomes in the “plan ahead” category, though with the difference that these behaviors have greater involvement of management and less intrusive clinical changes than those in the “increase efficiency” category. The “change the coding” category is different, however, and shows clear evidence of self-enhancing responses to performance measurement. Measuring clinical practices in ways that enhance the economic outcomes is a post hoc approach of improving the economic performance with no clinical changes, and one that results in improved measured performance and (in some reimbursement schemes) higher actual reimbursement.

 Reframing decision-making is the most interesting category of responses documented by Waitzberg et al because it demonstrates the problems in resolving tensions introduced by multiple goals. When the decision-makers emphasized averages rather than individual cases they were clearly marrying pursuit of the clinical goal for patients requiring high-cost procedures with self-enhancement on the economic dimension. A patient mix with sufficiently many low-cost procedures to maintain a low average draws attention away from the higher-cost procedures and hence allows doctors to offer clinical treatment holding the quality they see as crucial while avoiding management goal shortfalls on the economic dimension or management instructions to save money also on individual patients. It maintained clinical outcomes at a high level while compromising economic outcomes.

 When decision-makers developed and refined tools for decision-making, they appeared to make similar compromises, but with the economic goal taking a more prominent role. Limiting use of expensive diagnostic services and using multiple clinical specialties to discuss potential surgery, as reported by Waitzberg et al, are routinized ways of economizing on the resource use, and hence performing better on the economic dimension. An important feature of such tools of decision-making is that they are rule-like in nature and hence involve less individual decision-making. In the absence of such tools, the clinical decision-maker might choose a more expensive procedure to reduce risk as much as possible, but such individual risk-reduction decisions could accumulate costs to a level that is inconsistent with the economic goals of the clinic. Hence, rule-like tools for decision-making are introduced to give greater emphasis to economic goals while creating looser association of each clinical decision-maker with the clinical goals. Just as the averaging of clinical costs in the “working with averages” reframing allows pursuit of specific clinical goals at some economic cost, so does the averaging of risk in the “tools for decision-making” reframing allows lower economic cost at some risk of worse clinical outcomes. The clinical and economic goals interact in both cases, and the result is a compromise. The quality of the compromise along both goal dimensions is unclear, as it will depend on how adept the decision-makers are in employing their dual agency.

## Further Research on Hospitals’ Multiple Goals

 Together, the theory and evidence reported in Waitzman et al and the broader research stream on multiple goals noted in this article point to the importance of further research. Research on organizational search for solutions to performance below goal levels started with the assumption of sequential attention: goals are ordered by their importance for the organization, and the most important goal with low performance will be attended to first.^3^ This is a workable assumption in many contexts, but it is problematic for organizations that operate dangerous technologies and seek profitability.^9^ It is also a poor fit to hospitals, as they have an overarching goal of providing good clinical outcomes but also a necessary goal of being economically viable. Multiple goals that do not have a clear priority order is seen in many types of organizations, and the intrinsic nature of this goal structure to hospitals and other healthcare providers suggests that this is a context that could yield pioneering and important findings.

 Waitzberg et al have through their novel data and methodology provided important clues for future research, as noted above. Through their emphasis on individual decision-making, they have given a view of how this conflict unfolds at the most micro level. For completeness, this should be coupled with a macro-level view. It is well known that organizational structures and processes and individual action interact and adapt to each other. Documenting the adaptation from one side is valuable but begs the question of what the other side looks like. How do organizations differ in their measurement of performance and assignment of goals to individuals in either management or clinical roles? What are the consequences of these differences? Do organizations anticipate or react to the dual agency shown by individual decision-makers?

 From the viewpoint of theory and evidence on multiple goals, the following questions take a primary role. To what extent do the hospitals generate detailed information on the economic and clinical performance? To what extent do hospitals have numeric goals on the economic and clinical performance? To whom is each type of goal assigned, and how are aspiration levels for the desired performance level set? If economic goals and clinical goals and assigned to different people, how are they reconciled? If economic goals and clinical goals are assigned to the same person, how do they respond to different levels of success (or failure) on each goal dimension?

 This is a long string of specific question that individually will advance our knowledge of the reconciliation of multiple goals in organizations generally, and in hospitals specifically. Equally important, the knowledge generated from answering these questions holds the promise for improving the clinical and economic outcomes in healthcare, a sector of the economy that has an important societal role.

## Ethical issues

 Not applicable.

## Competing interests

 Author declares that he has no competing interests.

## Author’s contribution

 HRG is the single author of the paper.
